# Improvement of Antioxidant Defences and Mood Status by Oral GABA Tea Administration in a Mouse Model of Post-Stroke Depression

**DOI:** 10.3390/nu9050446

**Published:** 2017-04-29

**Authors:** Maria Daglia, Arianna Di Lorenzo, Seyed Fazel Nabavi, Antoni Sureda, Sedigheh Khanjani, Akbar Hajizadeh Moghaddam, Nady Braidy, Seyed Mohammad Nabavi

**Affiliations:** 1 Department of Drug Sciences, Medicinal Chemistry and Pharmaceutical Technology Section, Pavia University, VialeTaramelli 12, 27100 Pavia, Italy; arianna.dilorenzo01@universitadipavia.it; 2 KOLINPHARMA S.p.A., Lainate, Corso Europa 5, 20020 Lainate (MI), Italy; 3 Applied Biotechnology Research Center, Baqiyatallah University of Medical Sciences, P.O. Box 19395-5487, Tehran 19395-5487, Iran; Nabavisf@gmail.com; 4 Grup de Nutrició Comunitària i Estrès Oxidatiu and CIBEROBN (Physiopathology of Obesity and Nutrition), Universitat de les Illes Balears, Palma de E-07122 Mallorca, Spain; tosugo@hotmail.com; 5 Department of Physiology, Faculty of Biological Sciences, Shahid Behshti University, P.O. Box 19615-1178, Tehran 19615-1178, Iran; S.khanjani66@yahoo.com; 6 Department of Biology, Faculty of Basic Sciences, University of Mazandaran, 47416-95447 Babolsar, Iran; a.hajizadeh@umz.ac.ir; 7 Centre for Healthy Brain Ageing, School of Psychiatry, University of New South Wales, Sydney, NSW 2052, Australia; n.braidy@unsw.edu.au; 8 CHeBA NPI, Euroa Centre, Prince of Wales Hospital, Barker Street, Randwick, NSW 2031, Australia

**Keywords:** GABA green tea, GABA oolong tea, post-stroke depression, antioxidant activity

## Abstract

Green GABA (GGABA) and Oolong GABA (OGABA) teas are relatively new varieties of tea, whose chemical composition and functional properties are largely under-studied, despite their promising health capacities. Post stroke depression (PSD) is a complication of stroke with high clinical relevance, yielding increasing mortality and morbidity rates, and a lower response to common therapies and rehabilitation. Methods: Two chemically characterized commercial samples of GGABA and OGABA were investigated for effects on mood following oral administration using a mouse model of PSD, through common validated tests including the Despair Swimming Test and Tail Suspension Test. Moreover, the antioxidant activity of GGABA and OGABA was evaluated by determining the levels of lipid peroxidation products and the activity of antioxidant enzymes in the mouse brain in vivo. Results: GGABA and OGABA attenuated depressed mood by influencing behavioral parameters linked to depression. GGABA was more active than OGABA in this study, and this effect may be likely due to a higher content of polyphenolic substances and amino acids in GGABA compared to OGABA. GGABA also exerted a greater antioxidant activity. Conclusions: Our data suggests that GABA tea is a promising candidate that can be used as an adjuvant in the management of PSD.

## 1. Introduction

Stroke is a cerebrovascular event affecting one in 400 people each year worldwide. It represents the second leading cause of mortality, and one of the most common causes of long-term disability [1]. According to brain imaging studies, two different types of stroke can be recognized: ischemic stroke, which accounts for more than 80% of all cases and results from vessel occlusion due to thrombosis, embolysism or systemic hypoperfusion, and hemorrhagic stroke, caused by vessel rupture [2]. Known complications of stroke of high clinical relevance, due to their frequency and impact on both cognitive and physical outcomes for patients, include neuropsychiatric disorders in general and depression in particular. Post-stroke depression (PSD) affects one third of patients, manifesting between 4 weeks to 1 year after the cerebrovascular event, influencing mortality and morbidity rates, and reducing the effect of common therapies and rehabilitation [3]. Patients who develop PSD have significantly higher mortality rates than similarly impaired stroke patients who do not experience mood disorders [4,5,6]. The common therapeutic approach for the treatment of PSD is based on treatment with antidepressants, which appear to be efficient adjuvants towards reaching partial or full recovery and independence. On the other hand, antidepressants may cause various and serious adverse effects, especially in the elderly, which may lead to the suspension of treatment, compromising the healing process. 

Oxidative stress, defined as an imbalance between oxidant agents and endogenous and exogenous antioxidant defenses, plays a pivotal role in the development of ischemic damage, since it mainly occurs during the post-ischemic reperfusion stage. Many studies have identified potential peripheral biomarkers of oxidative stress in the biological fluids of stroke patients (i.e., blood, urine and cerebrospinal fluid). These biomarkers are mainly products of oxidative damage, which are not to be confused with reactive oxygen species (ROS). The latter are not suitable biomarkers due to their short half-life and high reactivity. For example, decreases in vitamin C and increases in thiobarbituric-reactive substances (TBARS) have been measured 2 days following the onset of stroke [7]. Another study highlights increased levels of malondialdehyde (MDA) and hydroxynonenal (HNE), two main products of lipid peroxidation, in ischemic stroke patients compared with healthy subjects [8]. Plasma levels of vitamin E, vitamin A, and uric acid were also reported to be lower in patients with ischemic stroke [9,10]. Taken together, this evidence highlights the clinical relevance of oxidative damage in the pathophysiology of ischemic stroke. 

A series of environmental, genetic, and medical factors can promote oxidative and nitrosative stress in depressed patient brain, which, in turn, stimulates the production of pro-inflammatory cytokines leading to neuro-inflammation. Both oxidative and nitrosative stress and neuro-inflammation can affect the development of depressive symptoms and promote cellular damages and dysfunctions, which might contribute to depressive symptoms and to the decrease of neurogenesis and neuroplasticity, promoting neuronal apoptosis. Moreover, the cellular damages can stimulate autoimmune and inflammatory pathways, which could lead to an increase of oxidative and nitrosative stress itself, reinforcing depressogenic effects [11].

An imbalance between oxidative stress biomarkers and antioxidants has also been identified in patients with depression. Several enzymes related to the production and cleavage of ROS, such as xanthine oxidase, superoxide dismutase (SOD), catalase and glutathione peroxidase, have been found to be lower in the blood of depressed patients when compared with non-depressed controls [12,13,14]. Higher serum levels of 8-hydroxy-2′-deoxyguanosine (a marker of oxidative DNA damage) and lower plasma levels of vitamins C and E have been found in depressed patients than in healthy subjects [15,16]. 

A growing body of evidence suggests that (i) oxidative stress could be recognized as an important pathophysiological agent common to both stroke and depression, and (ii) polyphenols and secondary plant metabolites may exert protective activity against oxidative stress in both stroke and depression. Our research group put forward the hypothesis that polyphenols could have a positive role in the management of post-stroke depression on the basis of their antioxidant properties [17,18,19]. 

Green and GABA green tea, are new types of tea with high levels of γ-aminobutyric acid which accumulate in tea leaves due to anaerobic fermentation under a nitrogen atmosphere [20]. The efficacy of both these tea beverages against PSD was subsequently demonstrated by our research group [21]. Furthermore, we have demonstrated that green tea and GABA green tea, administered intraperitoneally for one week at two different doses (50 mg/kg and 100 mg/kg) to experimental animals with induced PSD, are able to restore parameters linked to behavior, assessed through three common validated tests (i.e., the Despair Swimming Test -DST-, Tail Suspension Test -TST- and Open Field Test -OFT-). Moreover, both teas exerted an in vivo antioxidant effect, and were able to reduce the levels of lipid peroxidation products and to increase endogenous antioxidant defenses (i.e., SOD and catalase activities and GSH levels). Intraperitoneal administration is not a treatment representative of regular tea consumption, but was used to bypass gastroduodenal digestion, which induces significant changes in phytochemical composition [22]. Our data provides the first evidence of the in vivo antioxidant and antidepressive-like activities of green and GABA green tea, as well as their first exhaustive phytochemical profile. Since GABA green tea was found to be more active than common green tea, especially with regards to behavioral parameters, we have focused our attention on this particular type of tea. Thus, the aim of this study was to investigate the effects of orally administered, chemically characterized GABA green tea (GGABA) and GABA oolong tea (OGABA) extracts on the antioxidant and mood status on a mouse model of PSD. 

## 2. Materials and Methods 

### 2.1. Chemicals and Reagents

GABA green tea and GABA oolong tea were purchased from an Italian specialist tea shop (La Teiera Eclettica, Milan, Italy). Gallic acid, epigallocatechin-gallate, epicatechin-gallate, theanine, glutamic acid and glutamine were obtained from PhytolabGmbH and Co., KG (Vestenbergsgreuth, Germany). Bovine serum albumin and a kit for protein determination were purchased from ZiestChem Company (Tehran, Iran). In addition, 5,5-dithiobis(2-nitrobenzoic acid), ethylenediaminetetraacetic acid, nitro blue tetrazolium chloride, potassium dihydrogen phosphate, reduced glutathione, sodium dihydrogen phosphate, trichloroacetic acid, thiobarbituric acid, hydrogen peroxide, sodium carbonate, hydroxylamine chloride, ketamine, lidocaine, xylazine, FolinCiocalteau’s reagent, LC/MS-grade methanol and acetonitrile, formic acid solution 1 M, caffeine, epicatechin, catechin, sodium acetate, trimethylamine, tetrahydrofuran, ortho-phthalaldehyde, fluorenylmethylchloroformate were purchased from Sigma-Aldrich Chemical Company (St. Louis, MO, USA). Other chemical reagents and solvents were purchased from Merck Chemical Company, Darmstadt, Germany.

### 2.2. Preparation of GABA Tea Extracts 

GABA tea extracts were prepared as recommended by the supplier, under conditions commonly used for the infusion of tea. The aqueous extract was taken from a suspension containing 25 g of dried leaves in 500 mL of mineral water, whose mineral composition and content are reported in Table 1, at 85 °C for 2 min. After infusion the suspension was cooled at room temperature and filtered through a paper filter under vacuum. The aqueous extracts were submitted to centrifugation at 8000 rpm at room temperature for 10 min and the supernatant was filtered through a cellulose acetate/cellulose nitrate mixed esters membrane (0.45 μm; Millipore Corporation, Billerica, MA, USA). The extracts were subdivided into different aliquots and freeze-dried in order to be submitted to qualitative and quantitative analyses and pharmacological evaluation. 

### 2.3. Total Polyphenol Content

The total polyphenol content of both tea extracts was evaluated using the Folin-Ciocalteu’s method, with gallic acid used as standard [21]. In brief, 0.5 mL of Folin Ciocalteau’s reagent was added to 0.1 mL of each tea extract at a concentration of 1 mg/mL in water. After mixing, 2 mL of 15% Na_2_CO_3_ were added and the volume was brought up to 10 mL with Millipore-grade water. After mixing, the samples were kept in the dark for 2 h. After the reaction period, the absorbance was measured at 750 nm. Each tea sample was analyzed in triplicate and the concentration of total polyphenols was determined in terms of gallic acid equivalents (GAE), according to the following calibration curve: Absorbance (Abs) = 0.0017 concentration (μg/mL) + 0.0598 (*R*^2^ = 0.9984), obtained from analyses of gallic acid solutions ranging from 10 to 500 μg/mL.

### 2.4. RP-HPLC-PDA-ESI-MS^n^ Analysis

RP-HPLC-PDA-ESI-MS^n^ analysis was performed using a Thermo Finnigan Surveyor Plus HPLC, equipped with a quaternary pump, a Surveyor UV-Vis diode array detector, and a LCQ Advantage Max ion trap mass spectrometer (Thermo Fisher Scientific, Waltham, MA, USA), connected through an ESI source. A Zorbax Eclipse XDB-C18 column (150 × 4.6 mm, 5 μm), equipped with a Hypersil Gold C18 precolumn (10 × 2.1 mm, 5 μm), both from Agilent (Waldbronn, Germany), was used. The mobile phase contained water acidified with 0.1% formic acid (eluent A) and methanol (eluent B), and was eluted in a gradient as follows: from 10 to 70% B in 84 min, from 70% to 80% B in 5 min, from 80% to 100% B in 10 min, followed by a 5 min isocratic run of 100% B. Total run time was 105 min, including column reconditioning. The flow rate was maintained at 0.3 mL/min, the autosampler and column temperatures were maintained at 4 and 25 °C, respectively. Tea extracts were analyzed at a concentration of 5 mg/mL in water and 5 μL of the solution was injected into the chromatographic system. Chromatograms were registered at 210, 254, and 280 nm; spectral data were collected in the range of 200–800 nm for all peaks. 

HPLC-ESI-MS^n^ data were acquired under positive and negative ionization modes, using Xcalibur software. To achieve this, the ion trap operated in full scan (100–2000 *m*/*z*), data-dependent scan and MS^n^ modes. To obtain MS^2^ data, a 35% collision energy and an isolation width of 2 *m*/*z* were applied. A preliminary experiment was performed to optimize MS operating conditions: 5 μg/mL caffeine (0.1% formic acid and methanol, 50:50, % *v*/*v*) and 10 μg/mL (±)-catechin (0.1% formic acid and methanol, 50:50, % *v*/*v*) solutions were directly infused through the ESI interface at a flow rate of 25 μL/min into the mass spectrometer. The optimum conditions for the assay were the following: sheath gas 60, capillary temperature 220 °C, spray voltage 4.5, auxiliary gas 25 and 20, capillary voltage—47.20 V for the negative and 5 V for the positive ionization modes.

### 2.5. RP-HPLC-PDA Analysis 

The quantification of caffeine and flavan-3-ols in tea samples was performed through a RP-HPLC-PDA method developed and validated on a 1100 Agilent HPLC system (Agilent, Waldbronn, Germany) equipped with a gradient quaternary pump and a diode array detector. Agilent Chemstation software was used for HPLC system control and data processing. Separation was achieved on a Zorbax Eclipse XDB-C18 column (150 × 4.6 mm, 5 μm), equipped with a Hypersil Gold C18 (10 × 2.1 mm, 5 μm) precolumn, both from Agilent (Waldbronn, Germany). The mobile phase consisted of water acidified with 0.1% formic acid (eluent A) and methanol (eluent B) and was eluted in a gradient as follows: from 10 to 70% B in 84 min, from 70 to 80% B in 5 min, from 80 to 100% B in 10 min, followed by a 5 min isocratic run of 100% B. Total run time was 105 min, including column reconditioning. The flow rate was maintained at 0.5 mL/min, the autosampler and column temperatures were maintained at 4 and 25 °C respectively, and the injection volume was 5 μL. Spectral data were acquired between 190 and 600 nm for all peaks, while chromatograms were registered at 210, 254 and 280 nm. Caffeine, epicatechin (EC), catechin (C), epicatechin-3-gallate (ECG), epigallocatechin-3-gallate (EGCG) and gallic acid (GA) were identified by comparing their retention time and UV-Vis spectrum with that of commercial standards. 

### 2.6. RP-HPLC-PDA Method Validation 

Validation of the analytical method was performed following ICH guidelines [23]. Stock solutions of selected analytes (500 μg/mL) were prepared by dissolving 5 mg of each in 10 mL of 0.1% formic acid and methanol (50:50, % *v*/*v*) and stored at 4 °C until assay. A series of working solutions for GA and EC (7, 10, 25, 50, 100, 250, 300, 400 μg/mL) and for EGCG, ECG, caffeine and catechin (10, 25, 50, 100, 250, 300, 400 μg/mL) were prepared by diluting the stock solution with 0.1% formic acid and methanol (50:50, % *v*/*v*). 

Linearity was determined through the external standard method: for each compound, eight solutions at different concentrations, ranging from 25 to 500 μg/mL for catechin, from 7 to 500 μg/mL for GA and EC, and from 10 to 500 μg/mL for EGCG, ECG and caffeine, were analyzed in triplicate. The calibration curve for each analyte was obtained by plotting the mean peak areas (*y*) versus their nominal concentrations (*x*). Calibration curves (slope and intercept) and correlation coefficient (*R*^2^) were calculated as regression parameters by linear regression. 

The accuracy of this method was measured through a recovery assay, where spiked tea samples were analyzed at the same concentration levels as the standard concentrations. The study was performed in triplicate, and accuracy expressed as a percentage of the amount recovered compared to that from standard concentrations. Both intra-day (repeatability) and inter-day (intermediate precision) measurements were taken. Repeatability was investigated analyzing spiked tea samples in triplicate using three levels of concentration, corresponding to 25%, 50% and 100% of analytes. Intermediate precision was established using newly prepared solutions, using the same concentration levels as the repeatability study, over two successive days. Results are expressed as the relative standard deviation percentage of the measurements (RSD %). The sensibility of the method was evaluated as the limit of quantification (LOQ) and limit of detection (LOD). The LOD and LOQ were estimated using calibration curves calculated during the validation procedure, from which the average of the slope (S) and the standard deviation of the intercept (δ) were calculated. LOD and LOQ were obtained as follows: LOD = 3.3 δ/S, LOQ = 10 δ/S.

### 2.7. Determination of Glutamic Acid, Glutamine, γ-Amino Butyric Acid and Theanine

Amino acid analysis was performed as previously reported by Di Lorenzo et al. [21]. In detail, the analysis was performed using a Jasco X-LC system equipped with a 3159AS autosampler, 3185PU Xtreme high pressure pumps, a 3080DG degasser, a CO2060Plus column oven compartment, and a 3020FP fluorescence detector connected to a HP ProDesk G1 400 MT processor, Intel Core i5. Separation was achieved on Hypersil ODS (250 × 2.1 mm, 5 μm) thermostated at 40 °C. The mobile phase consisted of a solution of sodium acetate (20 mM), triethylamine (TEA, 0.018%–*v*/*v*) and tetrahydrofuran (THF, 0.3%–*v*/*v*), at pH 7.2 (eluent A) and an aqueous solution of sodium acetate (100 mM), methanol (40%) and acetonitrile (40%), at pH 7.2 (eluent B). The elution was performed in 17 min, using a gradient between 1 and 60% B, at a flow rate of 0.45 mL/min with an injection volume of 1 μL. The tea samples underwent gas-phase hydrolysis with HCL 6M prior to amino acid analysis. Hydrolysis was performed in glass sterilized tubes at 110 °C for 24 h under a nitrogen atmosphere. Free amino acids were then derivatized by ortho-phthalaldehyde (OPA) and fluorenylmethylchloroformate (FMOC) leading to the formation of derivatives from primary amino acids and secondary amino acids respectively. Derivatization was performed according to the Jasco autosampler program, preparing a mixture of sample:OPA:FMOC at a 1:1:1 ratio at room temperature (OPA and FMOC are CPS products). The derivatives were detected with a fluorometric detector (emission λ = 456 nm–excitation λ = 342 nm for OPA derivatives and emission λ = 272 nm–excitation λ = 312 nm for FMOC derivatives). Amino acid identification was performed via elution times of the obtained derivatives and compared to a mixture of standard amino acids submitted to derivation in identical test conditions.

### 2.8. Animals 

Male balb/c mice (5 weeks old, weighing between 20–25 g), were bought from the amol branch of the Pasteur Institute of Iran. All animals were kept in an appropriate room at 24 ± 2 °C under a light/dark 12/12 h cycle and 60% ± 5% humidity. Food and water were supplied ad libitum. The mice were acclimated to the test room for at least 24 h before the behavioral assay. In this study, behavioral examination was performed between 10.00 and 14.00 h. The animal experiments were processed following internationally accepted ethical guidelines for the care of laboratory animals in accordance with Principles of Laboratory Animals Care (NIH Publication No. 85-23, revised 1996). The ethical approval number is “81/021, 10 July 2002”.

### 2.9. Induction of Stroke

Ischemic stroke was induced in the mice after anesthesia with ketamine (60 mg/kg i.p.) and xylazine (5 mg/kg i.p.). Bilateral occlusion of the common carotid artery (BCCAO) was done according to standard experimental animal procedure for ischemic stroke. Briefly, the right and left carotid arteries were located and held over 5 min with vascular clamps (time under ischemia). Subsequently, the vascular clamps were removed for the following 10 min (time of reperfusion), and both carotid arteries were again restrained for 5 min. Finally, the vascular clamps were removed in order to allow the blood circulation to return to both carotid arteries. Lidocaine was used as local anesthesia to suture the surgical incisions and the area was rinsed with an antiseptic solution. All animals were housed in separated cages at room temperature until body temperature was recovered. Rectal temperature was checked daily and animals at 37 ± 1 °C were included in the experiment. Animals reporting abnormal behavior, diarrhea or seizures were discarded [24]. 

### 2.10. Tea Extract Administration

Animals were randomly classified into 6 groups of 10 animals each. GABA green tea and GABA oolong tea were gavaged at 10 mg/kg or 20 mg/kg for a week, starting from the first day after induction of stroke. Animal weights were recorded daily to ensure precise dosage and a 20-gauge gavage needle was used for the administration of extracts. Depressive-like behavior was evaluated in all animals 30 min after the last application, measuring despair swimming and tail suspension tests. 

### 2.11. Examination of Stroke-Induced Anhedonia

For the examination of stroke-induced anhedonia, water bottles were removed from animal cages for a period of 6 h. Subsequently, two bottles were introduced to each cage. The first contained a 2% *w*/*v* sucrose solution while the second contained only water. This experiment focuses on evaluating the differences in quantity of sucrose and water consumed. The sucrose solution and water consumption were measured over 6 h [25]. 

### 2.12. Despair Swimming Test (DST) and Tail Suspension Test (TST)

The DST is a very common and standardized model for assessing depressive-like behavior in experimental animals. In brief, the mice were individually placed in an open cylinder (25 cm high and 10 cm diameter) filled with a 19 cm depth of fresh water at room temperature. The mice were left to swim for 6 min, analyzing the time of swimming, periods of immobility (the time spent without horizontal movement, simply retaining the head above the water surface), and climbing (the time in which animals try to maintain their forelegs above the water surface through active vertical movement). 

The TST is another widespread assay system which is used for examining depressive-like behavior of animals. In brief, the experimental animals were suspended at a height of 58 cm for 5 min, using sticky tape placed 1 cm from the tail tip. Immobility time was calculated as the time without any movement for the 5 min monitoring period [26]. 

### 2.13. Anesthesia and Tissue Collection

After the behavioral assay and 12 h starvation, the animals were anesthetized through administration of a ketamine (60 mg/kg, i.p.) and xylazine (5 mg/kg, i.p.) mixture. The entire brain was carefully dissected and held at −60 °C prior to biochemical analysis.

### 2.14. Preparation of Tissue Homogenate

All brain tissue from each mouse was homogenized in 1 mM EDTA containing 100 mM ice cold phosphate buffered saline (1:10, % *w*/*v*) pH 7.4, and centrifuged for 30 min at 12,000× *g* and 4 °C. The upper layer was recovered and utilized for biochemical assays.

### 2.15. Measurement of Protein Content

Protein levels were assayed in brain homogenates by employing the Bradford method with bovine serum albumin as standard [27].

### 2.16. Estimation of Lipid Peroxidation, Reduced Glutathione Level, Superoxide Dismutase and Catalase Activities 

Thiobarbituric acid reactive substance (TBARS), a marker of lipid peroxidation, was evaluated following the method developed by Esterbauer and Cheeseman [28]. Superoxide dismutase activity (SOD) was determined as according to the method developed by Misra and Fridovich [29]. Catalase activity was determined by following the method used by Bonaventura et al. [30] with slight modifications [21]. The levels of reduced glutathione (GSH) were evaluated using Ellman’s method [31].

### 2.17. Statistical Analysis

Statistical analysis was performed with a statistical package for the social sciences (IBM SPSS 21.0 for Windows, Chicago, IL, USA). The data were assessed using a Shapiro-Wilk W test to determine the normal distribution. Statistical significance of the data was determined by one-way analysis of variance (ANOVA). When significant differences were found, Bonferroni’s post-hoc analysis was used to establish the relationships between groups. Data was reported as a mean ± standard deviation (SD), and *p* < 0.05 was considered statistically significant. 

## 3. Results

GABA green tea (GGABA) and GABA oolong tea (OGABA) extracts were prepared, starting from commercial samples and following the instructions provided by the supplier, to mimic the common conditions used for the preparation of tea beverages. The beverages were freeze-dried and the dry residue was assessed at 11.2 mg/mL and 7.2 mg/mL for GGABA and OGABA, respectively. Both extracts were submitted to RP-HPLC-PDA-ESI-MS^n^ analysis to determine their metabolite profiling; to RP-HPLC-PDA and RP-HPLC-FD analysis to quantify some representative components and amino acids, and to in vivo tests, to assess their antioxidant activity and antidepressive-like activity in a mouse model of PSD. 

### 3.1. RP-HPLC-PDA-ESI-MS^n^ Analysis

**Figure 1:**
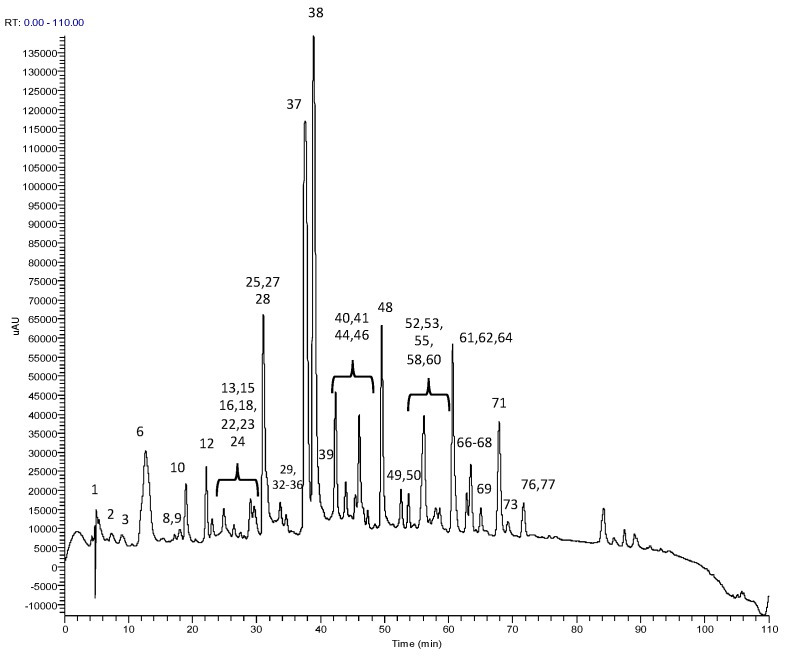
Chromatogram, acquired as total scan Photodiode Array (PDA) Detector, of Oolong GABA (OGABA) extract at 5 mg/mL.

To the best of our knowledge, the metabolite profiles of GABA oolong tea were yet to be studied. So, the metabolite profiling of GGABA and OGABA extracts were determined through RP-HPLC-PDA-ESI-MS^n^ analysis, which allowed for the identification of 53 compounds, listed in Table 2. The identification was performed through comparison of experimental data (chromatographic behavior, UV-Vis, MS and MS^n^ spectra, Table 2) with that available in the literature. Figure 1 shows, as an example, a OGABA chromatogram, acquired as total scan PDA. 

The identified compounds consisted of: (a) two xanthines (caffeine and theobromine); (b) six organic and phenolic acids (gallic acid, quinic acid, galloylquinic acid, 3-*O*-*p*-coumaroylquinic acid, 4-*O*-*p*-coumaroylquinic acid and 5-*O*-*p*-coumaroylquinic acid); (c) six flavones (6-*C*-arabynoil-8-*C*-glucosyl apigenin, 6-*C*-glucosyl-8-*C*-arabynoil apigenin, di-pentosylapigenin, rhamnosyl-hexosylapigenin, and two isoforms of di-hexosylapigenin); (d) eight flavan-3-ols (gallocatechin, epigallocatechin, catechin, epicatechin, epicatechingallate, epigallocatechingallate, (epi)catechin-3-*O*-(3-*O*-methyl)-gallate, and (epi)-afzelechin); (e) four theaflavins (theaflavin, theaflavindigallate, and two isoforms of theaflavingallate); (f) 14 tannins (galloylglucose, digalloylglucose, trigalloylglucose, strictinin, prodelphinidin, prodelphinidingallate, (epi)afzelechingallate-(epi)catechingallate, (epi)catechin-(epi)gallocatechingallate, theasinensin A, theasinensin B, theasinensin C, three isoforms of procyanidin and two isoforms of procyanidingallate); and (g) 10 flavonols (myricetinhexoside, myricetinhexosylrutinoside, quercetinhexoside, quercetinhexosylrutinoside, quercetinrutinoside, kaempferolhexoside, kaempferolrutinoside, two isoforms of kaempferolhexosylrutinoside, and kaempferol 3-*O*-*p*-coumaroyl-dirhamnosyl-hexoside). 

### 3.2. RP-HPLC-PDA Method Validation and Bioactives Quantification

The quantification of GA, caffeine, EGCG, ECG, EC and catechin in GGABA and OGABA extracts was performed by the standard addition method and expressed as μg/mL of tea beverage, taking into account tea dried residue after freeze-drying. Prior to quantification, the developed RP-HPLC-PDA method was validated following the ICH guidelines [23]. For each analyte, the concentration range, the calibration curve and the correlation coefficient (*R*^2^) were calculated and are reported in Table 3. The method was linear within the following ranges: 25 to 500 μg/mL for catechin, from 7 to 500 μg/mL for GA and EC, and from 10 to 500 μg/mL for EGCG, ECG and caffeine, and the correlation coefficients were all higher than 0.999. 

To evaluate the accuracy and precision of the method, one spiked tea sample was analyzed at three different concentration levels on three different days. Accuracy, intra-day and inter-day precision values are reported in Table 4. The results obtained indicate that the developed method was accurate, providing recoveries ranging from 86.1% to 106.3%, and precise, since the intra-day and inter-day variation were lower than 1% and 5% for all the analytes, respectively. 

As far as sensibility is concerned, LOQ and LOD values determined for each analyte are listed in Table 4. The very low concentrations indicate that the RP-HPLC-PDA method was very sensible for both quantification and detection purposes. 

The validated RP-HPLC-PDA method was applied to the quantification of catechin, epicatechin, ECG, EGCG, GA and caffeine in GGABA and OGABA extracts. The results, reported in Table 5, showed that GGABA tea is richer in flavan-3-ols and caffeine than OGABA. Moreover, OGABA, as expected showed a higher content of gallic acid deriving from the oxidative reactions, which gallate esters of flavan-3-ols undergo during the fermentation process. 

### 3.3. RP-HPLC-FD Analysis

**Figure 2:**
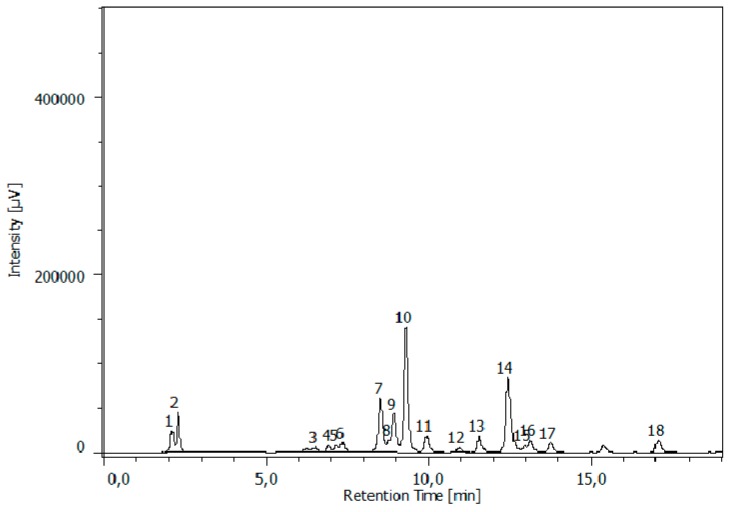
HPLC/FLD chromatograms of GGABA extract at the concentration of 0.5 mg/mL, respectively. The analysis showed the presence of (1) aspartic acid; (2) glutamic acid; (3) serine; (4) glutamine; (5) glycine; (6) threonine; (7) alanine; (8) arginine; (9) GABA 1; (10) theanine; (11) tyrosine; (12) cysteine; (13) valine; (14) GABA 2; (15) phenylalanine; (16) isoleucine; (17) leucine; (18) proline.

The determination of proteic and non-proteic amino acids was performed by means of a RP-HPLC-FD analysis, preceded by pre-column derivatization of amino acids. Their identification was based on the chromatographic behavior of amino acid derivatives using derivatised commercial standards as a reference. Figure 2 reported, as an example, the chromatogram of GGABA extract.

The analysis revealed the presence of 18 amino acids, with GABA, glutamine, glutamic acid and theanine quantified among these by the standard addition method. As reported in Table 5, GGABA extract showed a higher amount of all quantified amino acids, with the exception of GABA content which was higher in the OGABA extract.

### 3.4. Antidepressive-Like Activity and In Vivo Antioxidant Effects of GGABA and OGABA Extracts

**Figure 3:**
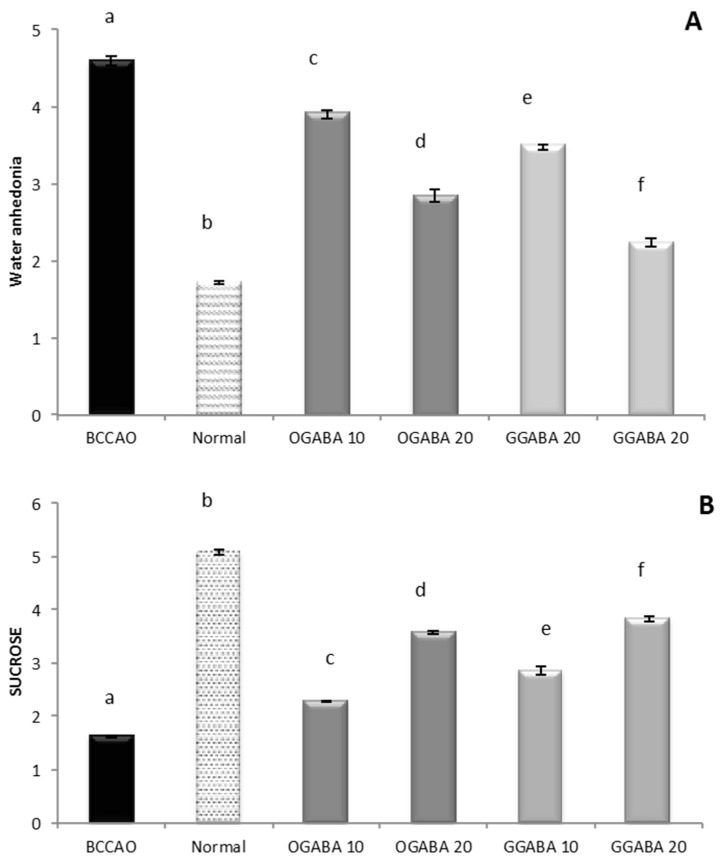
Volume of water (**A**) and sucrose solution (**B**) consumption in stroke-induced anhedonia model. Data are shown as a mean (mL) ± SD (*n* = 3); different letters indicate statistically significant differences (*p* < 0.05) between the two groups.

In this study, animals were divided into 4 major groups: (a) a control group containing healthy non ischemic mice; (b) a BCCAO group, made up of mice in which ischemic stroke was induced through bilateral common carotid artery occlusion (BCCAO) and which did not receive any treatment; (c) 2 groups treated with GABA green tea (GGABA) extract at two dosages (10 and 20 mg/kg), after the induction of ischemic stroke; and (d) 2 groups treated with GABA oolong tea (OGABA) extract at two dosages (10 and 20 mg/kg) after surgery. Tea extracts were administered through oral gavage, to mimic the conventional way of drinking tea. This administration route makes it possible to evaluate the in vivo activities of tea following gastroduodenal digestion, which is known to cause deep changes in the chemical composition of tea [22], and the hepatic first pass effect. Mouse anhedonia, is defined as a loss of ability to experience satisfaction and is considered a central clinical trait of mental illness, including depression. Anhedonia is assessed in experimental animals by measuring the volume of consumption of both water and sucrose. As shown in Figure 3, the BCCAO group showed a significant decrease (*p* < 0.05) in sucrose consumption and a significant (*p* < 0.05) increase in water consumption compared to the control group, highlighting that our murine model is suitable for the study of PSD. The oral administration of both GGABA and OGABA extracts mitigates mice anhedonia, producing an increase in sucrose consumption and a decrease in water consumption. In both cases, the highest effect was registered at the highest dose, with GGABA being more active at both doses. 

**Figure 4:**
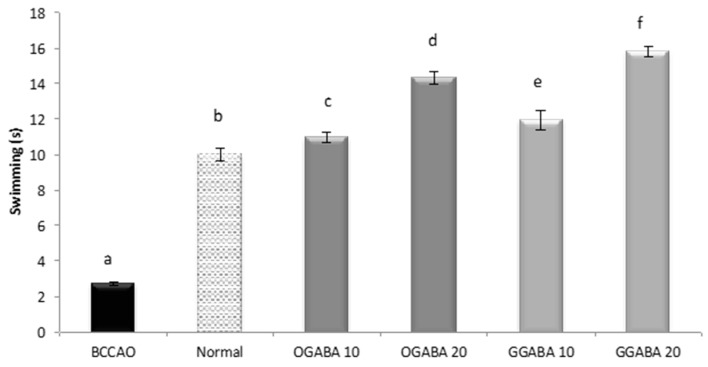
Effects of oral administration of OGABA and GGABA extracts on swimming time in forced swimming model. Data are mean(s) ± SD (*n* = 7); different letters indicate statistically significant differences (*p* < 0.05) between the two groups.

**Figure 5:**
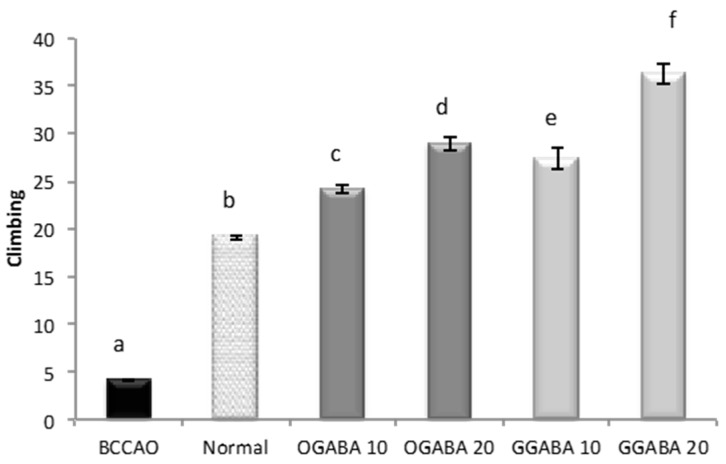
Effects of oral administration of OGABA and GGABA extracts on climbing time in forced swimming model. Data are mean(s) ± SD (*n* = 7); different letters indicate statistically significant differences (*p* < 0.05) between the two groups.

**Figure 6:**
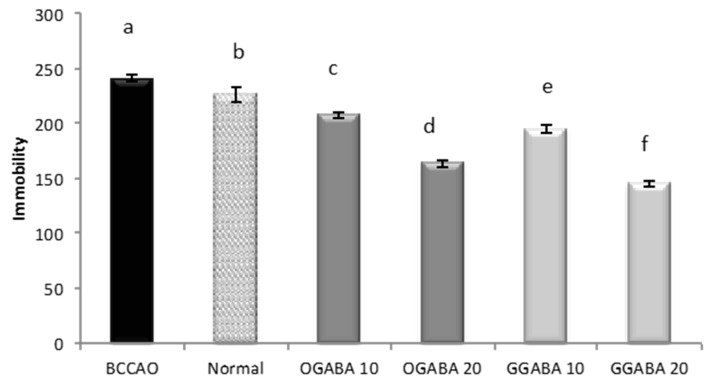
Effects of oral administration of OGABA and GGABA extracts on immobility time in forced swimming model. Data are mean(s) ± SD (*n* = 7); different letters indicate statistically significant differences (*p* < 0.05) between the two groups.

GGABA and OGABA were tested for antidepressant behavior using two common, validated tests: the despair swimming test (DST) and the tail suspension test (TST). All behavioral assessments were performed 30 min after the final administration. In DST, mice are forced to swim in a cylinder full of water. The test lasts 6 min, during which three parameters are registered: immobility, swimming and climbing times. For all three parameters (Figure 4, Figure 5 and Figure 6), a significant statistical difference was registered between BCCAO and control groups (*p* < 0.05), confirming that the selected animal model is suitable for the evaluation of PSD.

At both concentrations, tea extracts showed good antidepressive-like activity, since they were able to restore and even improve the normal behavior. In fact, tea administration significantly (*p* < 0.05) increase mobility periods (swimming times, Figure 4, and climbing times, Figure 5) and decrease immobility time (Figure 6). In all cases, GABA green tea was the most effective in reducing behavioral abnormalities, especially at the highest dose (20 mg/kg). 

**Figure 7:**
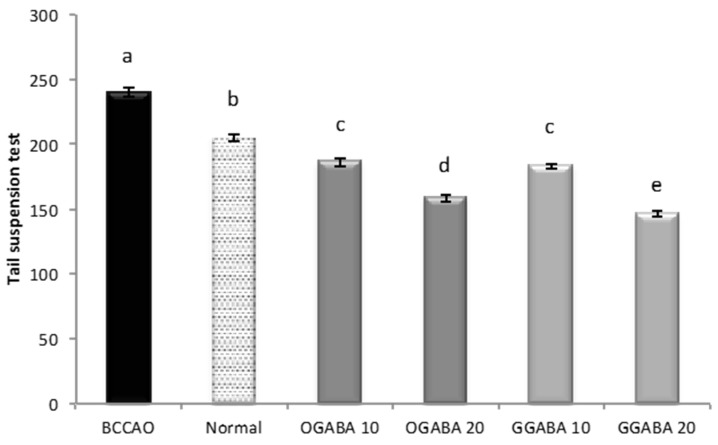
Effects of oral administration of OGABA and GGABA extracts on immobility time in tail suspension model. Data are mean(s) ± SD (*n* = 7); different letters indicate statistically significant differences (*p* < 0.05) between the two groups.

TST is a common behavioral test used for the evaluation of new potential antidepressant drugs in unavoidable and unescapable stress conditions. During the 6 min of test duration, one parameter (immobility time) is registered, which is reduced effective antidepressant agents. As shown in Figure 7, the oral administration of GGABA and OGABA extracts produced a significant (*p* < 0.05) reduction of the immobility time, with GGABA and OGABA showing the same activity (*p* > 0.05) at the lowest dosage (10 mg/kg). At the highest concentration (20 mg/kg), GGABA was once more found to be the most active. In our previous review article, we formulated the hypothesis that polyphenols could assume a positive role in the management of PSD, due to their antioxidant activity [19]. 

Our previous research on tea revealed that the intraperitoneal administration of both Chun Mee green tea and GABA green tea produced a mitigation of oxidative stress in mice brain [21]. TBAR levels were lower in brain homogenates of treated mice than in the control group, with GABA green tea successfully restoring normal values at the higher concentration tested (100 mg/kg). SOD activity and GSH levels were found to be higher, though complete restoration to normal, healthy values has not been registered [21]. Therefore, in this study we wanted to establish if this in vivo antioxidant activity of GABA tea is maintained with oral administration. 

**Figure 8:**
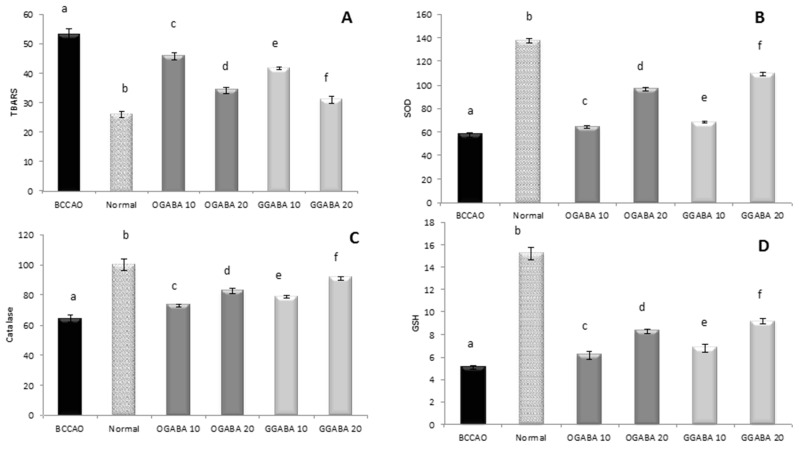
Effects of oral administration of OGABA and GGABA extracts on oxidative stress levels. In detail, thiobarbituric-reactive substances (TBARS) levels, expressed as nmol malondialdehyde (MDA) eq/g tissues (**A**); superoxide dismutase (SOD) activity, expressed as U/mg protein (**B**); Catalase activity, expressed as U/mg protein (**C**); and glutathione (GSH) levels, expressed as µg/mg protein (**D**) in mouse brain tissue are reported. Data are mean ± SD (*n* = 7); different letters indicate statistically significant differences (*p* < 0.05) between the two groups.

Lipid peroxidation was estimated from the TBAR levels, and taken for BCCAO, control and treated groups (Figure 8). As expected, stroke induced intense oxidative stress in mouse brain producing a significant (*p* < 0.05) increase in TBAR levels compared to control group. The administration of different dosages of OGABA and GGABA extracts produced a significant reduction in lipid peroxidation products, with GGABA being most active at both doses. The effect exerted by the highest dose was found to be highest. Anyway, the highest dosage was still unable to restore normal levels. A similar data trend can be seen in endogenous antioxidant defenses. BCCAO is characterized by lower levels of SOD and catalase activity and lowered GSH levels compared to healthy mice, indicating the presence of intense cerebral oxidative stress in mice affected by stroke. The administration of OGABA and GGABA extracts increase the antioxidant defenses, with GGABA being the most active. Even so, at the highest concentration tested (20 mg/kg) GGABA was not able to restore normal GSH levels and normal SOD and catalase activities. 

To evaluate a potential correlation between oxidative stress and mouse behavior, we decided to determine Pearson's linear correlation between parameters linked to mood status and oxidative stress biomarkers. As shown in Table 6, the coefficients highlighted that oxidative stress biomarkers and mood parameters are well related. Of all the possible combinations, the TBAR levels and Cat activities were most closely related to mood parameters. TBAR levels showed a moderate negative correlation with swimming and climbing times: the higher the TBARs, the lower the animals' mobility. However, TBAR levels showed a moderate positive correlation with the immobility times registered in both tests. Catalase activity showed the opposite correlation with both mobility and immobility times. 

## 4. Discussion

This study demonstrates that the oral administration of GGABA and OGABA extracts to experimental animals, in which PSD has been induced by BCCAO, (1) mitigates depressed mood, being able to improve behavioral parameters linked to depression, and (2) exerts in vivo antioxidant activity, being able to decrease lipid peroxidation product levels and increase SOD and catalase activities and GSH levels in mouse brain. GGABA, which was found to be the most active, exhibited higher caffeine, proteic and non proteic amino acid, and polyphenolic content, which could justify the highest capacity to influence mood status and antioxidant activity of this kind of tea. In particular, GGABA has twice the amount of caffeine and theanine in comparison with OGABA. A number of studies showed that when administered together, theanine and caffeine combination increases alertness, attention, and memory and influence positively mood status [32,33]. GGABA showed higher content of glutamic acid and glutamine, but these compounds probably do not exert any effect at brain level. In fact, as far as glutamic acid is concerned, several investigations have shown that the blood brain barrier (BBB) is impermeable to glutamate, even at high concentrations [34]. In addition, large amounts of glutamate administered to experimental animals and humans resulted to induce very small changes in glutamate plasma concentration in view of the fact that it is almost completely metabolized to produce energy. As regards glutamine, it is mainly deaminated by phosphate-dependent glutaminases to glutamate, which in turn is used as a source of energy [35,36]. As far as GABA is concerned, OGABA showed a slightly higher concentration (about 20%) than that found in GGABA. It is not easy to draw a conclusion about the influence of GABA considering that literature data regarding the GABA ability to cross the BBB are often contradictory, especially because of the different methods of administration (i.e., oral versus injection), and species used for the experiments [37]. In recent years, Boonstra et al. have speculated that GABA of food origin does exert a direct effect on central nervous system, being unable to cross the BBB, but acts on mood with an indirect mechanism of action via the enteric nervous system [37]. Finally, GGABA showed a polyphenolic content about ten times greater than that determined in OGABA, thereby justifying its higher in vivo antioxidant activity. In addition, Pearson’s correlation coefficients highlighted a relationship between mouse behavioral parameters and oxidative stress biomarkers, confirming our initial hypothesis, and showing that these biochemical markers could be good predictive parameters of depression in preclinical studies, to be confirmed by clinical trials. The obtained results were in agreement with those reported in our previous paper [21] and provided by Schuch et al. [38], which reported that TBARS serum levels are higher in severely depressed patients and showed that exercise, which reduces depression, also reduces serum TBARS levels after 3 weeks of treatment. 

This investigation also represents the first study of metabolite profiling of GABA oolong tea, detecting 48 compounds. The results from chemical analyses have revealed that the composition of GGABA and OGABA extracts is different, with OGABA being more complex than GGABA. The main differences are in the presence of tannins (i.e., flavan-3-ols dimers, theasinensins and procyanidins) and theaflavins. It is interesting to notice that some flavan-3-ols (i.e., epigallocatechin and (epi)catechin-3-*O*-(3-*O*-methyl)-gallate) were only detected in the OGABA extract.

While further efforts must be made to elucidate the molecular mechanisms underlying the antidepressive-like and in vivo antioxidant activities of GABA tea and the pharmacokinetics of GABA tea components, the varying effectiveness of GGABA and OGABA could be attributed to the higher amount of polyphenols, theanine and glutamine found in GGABA. 

## 5. Conclusions

In conclusion, this investigation showed that green and oolong GABA teas were able to exert positive effects on mood following oral administration using the mouse model of PSD, being green GABA tea more active than oolong GABA tea. In addition, these teas showed *in vivo* antioxidant activity, being able to decrease the levels of lipid peroxidation products and increase the activity of antioxidant enzymes in the mouse brain. The chemical composition of these teas, at least in part, explains the higher antidepressive-like and antioxidant activities of green GABA tea, which resulted to be richer in bioactive compounds than oolong GABA tea (i.e polyphenolic substances and amino acids). Taken together, the results obtained here and in our previous investigation suggest that GABA tea is a promising candidate to be used as an adjuvant in the management of PSD.

## Figures and Tables

**Table 1:** Composition of water utilized for tea infusion, expressed as mg of anion or cation dissolved in 1 L of water.

Anions/Cations	mg/L
HCO_3_^−^	7.5
F^−^	<0.1
NO_3_^−^	1.1
SO_4_^2−^	3.8
Ca^2+^	2.4
Na^+^	1.7
Total dissolved solids	23.8

**Table 2:** Chromatographic behavior (retention time, RT), UV, MS and MS/MS data of the compounds identified in GGABA and OGABA extracts.

Peak	RT (min)	λ _max_ (nm)	*m*/*z*	HPLC-ESI-MS/MS *m*/*z* (% of Base Peak)	Proposed Structure
**Xanthines**					
8	22.5	222, 273	181 ^+^	137 (100)	theobromine ^a,b^
24	38.7	230, 270	195 ^+^	138 (100)	caffeine ^a,b^
**Organic and phenolic acids**					
1	5.2	211	191	127 (100), 85 (80)	quinic acid ^a,b^
4	13.6	219, 274	343	191 (100), 169 (10), 125 (2)	galloylquinic acid ^a,b^
11	27.5	219, 270	169	125 (100)	Gallic acid ^a^
19	34.1	211, 330	337	163 (100), 191 (10), 119 (10), 173 (5)	3-*p*-coumaroylquinic acid ^a,b^
27	43.5	211, 310	337	191 (100), 173 (25), 163 (10)	5-*p*-coumaroylquinic acid ^a,b^
29	46.3	211, 309	337	173 (100), 163 (15), 191 (10)	4-*p*-coumaroylquinic acid ^a,b^
**Flavones**					
30	47.7	210, 270, 333	593	473 (100), 353 (25), 383 (15), 503 (30)	dihexosyl-apigenin ^a,b^
32	52.9	210, 275	563	503 (70), 473 (100), 443 (80), 383 (45), 353 (50)	6-*C*-arabinosyl-8-*C*-glucosyl apigenin ^a,b^
33	54.2	210, 275	563	503 (30), 473 (100), 443 (100), 383 (40), 353 (40)	6-*C*-glucosyl-8-*C*-arabinosyl apigenin ^a,b^
38	58.6	211, 258, 352	577	413 (100), 293 (30), 457 (10), 353 (2)	rhamnosyl-hexosylapigenin ^a^
39	59.08	211, 271, 336	593	413 (100), 293 (10), 473 (10)	dihexosyl-apigenin ^a,b^
42	60.6	256, 353	533	443 (100), 473 (50), 353 (20)	6,8-*C*-dipentoside apigenin ^b^
**Flavan-3-ols**					
6	19.5	220, 270	305	261 (40), 221 (85), 219 (75), 179 (100), 165 (35), 125 (20)	gallocatechin ^a,b^
15	31.3	240, 270	305	261 (40), 179 (100), 219 (70), 165 (35) 221 (85), 125 (25)	epigallocatechin ^a,b^
16	32.4	211, 278	289	245 (100), 205 (40), 179 (15), 203 (40), 109 (5)	catechin ^b^
25	39.2	233, 274	457	331 (70), 169 (100), 305 (35)	epigallocatechingallate ^a,b^
26	43.1	232, 278	289	245 (100), 205 (40), 109 (5), 137 (10), 125 (2)	epicatechin ^a,b^
31	52.8	230, 276	441	289 (100), 331 (20), 169 (25)	epicatechin-gallate ^a,b^
35	55.8	219, 270, 352	455	289 (100), 183 (25)	epicatechina-3-*O*-(3-metil)-gallato ^a^
37	57.2	209, 270	425	273 (100), 169 (40), 125 (5)	epiafzelechin-gallate ^a,b^
**Theaflavins**					
49	84.8	223, 270, 364, 470	867	715 (65), 697 (100), 679 (15), 527 (45), 545 (25), 559 (20)	theaflavin-digallate ^a^
50	84.9	213, 269, 367, 478	715	527 (100), 545 (80), 563 (70), 501 (40), 407 (10)	theaflavin-gallate ^a^
51	85.8	215, 270, 364	563	545 (100), 527 (30), 519 (55), 501 (30), 407 (70), 379 (50)	theaflavin ^a^
52	86.6	213, 269, 367, 478	715	527 (30), 545 (60), 563 (100), 501 (20), 407 (60)	theaflavin-gallate ^a^
**Flavonols**					
34	54.5	210, 273	787	316 (100), 769 (30), 359 (20), 625 (15), 725 (20)	myricetinhexosylrutinoside ^b^
36	55.9	215, 266, 350	479	316 (100), 317 (30)	myricetinhexoside ^a,b^
41	60.4	211, 256, 356	771	301 (100), 609 (10), 463 (2)	quercetinhexosylrutinoside ^a,b^
43	62.7	207, 267, 356	463	301 (100)	quercetinhexoside ^a,b^
44	63.1	211, 256, 357	609	301 (100), 271 (15)	quercetinrutinoside ^a,b^
45	64.5	210, 267, 348	755	285 (100)	kaempferolhexosylrutinoside ^a,b^
46	67.3	210, 267, 348	755	285 (100)	kaempferolhexosylrutinoside ^a,b^
47	70.7	206, 268	447	285 (40), 284 (100), 327 (20)	kaempferolhexoside ^a,b^
48	70.7	207, 266, 345	593	285 (100)	kaempferolrutinoside ^a^
53	96.3	220, 270	885	739 (100), 431 (20), 285 (10)	kaempferol-3-*O*-*p*-coumaroyl-dirhamnosyl hexoside ^a^
**Tannins**					
2	7.71	216, 267	609	471 (100), 591 (80), 565 (20), 525 (30)	theasinensin C ^a^
3	9.6	210, 260	331	169 (100), 271 (80), 211 (40), 193 (20), 125 (15)	galloylglucose^a,b^
5	18.1	211	609	483 (30), 441 (100), 423 (70), 305 (20), 591 (29)	prodelphinidin ^a,b^
7	22.0	211, 275	761	609 (100), 423 (80), 305 (20), 591 (70)	prodelphinidingallate ^b^
9	22.8	217, 270	761	609 (40), 591 (100), 453 (10)	theasinensin B ^a^
10	24.4	211, 278	577	425 (100), 407 (40), 289 (10), 451 (25)	procyanidin ^a^
12	29.4	210, 254	865	739 (15), 695 (100), 577 (45)	(epi)afzelechingallate-(epi)catechingallate ^a^
13	29.5	226, 275	633	301 (100), 463 (15)	strictinin ^a,b^
14	29.9	211, 278	577	425 (100), 407 (40), 289 (10), 451 (25)	procyanidin ^a,b^
17	32.5	223, 271	913	743 (100), 761 (50), 591 (80), 573 (45)	theasinensin C ^a^
18	34.0	221, 275	483	271 (100), 331 (20), 169 (10)	digalloylglucose ^b^
20	34.2	211, 276	745	559 (65), 407 (100), 619 (90), 577 (65), 441 (40)	(epi)catechin-(epi)gallocatechingallate ^a^
21	35.1	211, 278	577	425 (100), 407 (40), 289 (10), 451 (25)	procyanidin ^a,b^
22	35.9	211, 277	729	559 (100), 577 (95), 407 (20), 441 (5) 603 (25), 451 (35), 711 (15), 289 (10)	procyanidingallate ^a,b^
23	38.4	211, 277	729	407 (80), 577 (80), 711 (20), 559 (100), 28451 (50), 603 (50), 441 (50), 289 (10)	procyanidingallate ^a,b^
28	45.8	220, 280	635	465 (100), 483 (70), 313 (20)	trigalloylglucose ^b^

^+^ Compounds revealed in positive ionization mode; ^a^ compounds identified in GABA oolong tea extract; ^b^ compounds identified in GABA green tea extract.

**Table 3:** Concentration ranges, calibration curves, correlation coefficients of the RP-HPLC-PDA method for gallic acid (GA), caffeine, catechin, epigallocatechin-3-gallate (EGCG), epicatechin-3-gallate (ECG) and epicatechin (EC).

Analyte	Concentration Range (μg/mL)	Calibration Curve	*R*^2^
GA	7–500	*y* = 18.941*x* − 41.578	0.9999
caffeine	10–500	*y* = 19.254*x* − 49.677	0.9999
catechin	25–500	*y* = 5.4294*x* − 84.198	0.9998
EGCG	10–500	*y* = 9.6778*x* − 51.975	0.9999
ECG	10–500	*y* = 14.997*x* − 63.054	0.9999
EC	7–500	*y* = 5.6054*x* − 13.496	0.9999

**Table 4:** Accuracy (recovery %), intraday precision (RSD %), interday precision (RSD %), limit of quantification (LOQ) and limit of detection (LOD) of the analytical method, suitable for the quantification of GA, caffeine, catechin, EGCG, ECG and EC in tea samples. The concentration (µg/mL) of each analyte added to tea samples is reported.

	µg/mL	GA	µg/mL	Caffeine	µg/mL	Catechin	µg/mL	EGCG	µg/mL	ECG	µg/mL	EC
recovery (%)	2.75	105.7	12	100.6	5	87.3	10	90.7	5	91.1	3.75	95.9
	5.5	106.3	24.5	101.4	10	86.1	20	89.2	10	88.7	7.5	93.2
	11	103.1	49	101.0	20	87.5	40	87.8	20	86.4	15	91.3
Intraday precision (RSD %)	2.75	0.5	12	0.5	5	0.7	10	0.1	5	0.3	3.75	0.5
	5.5	0.3	24.5	0.1	10	0.2	20	0.1	10	0.3	7.5	0.3
	11	0.8	49	0.1	20	0.9	40	0.3	20	0.1	15	0.2
Interday precision (RSD %)	2.75	1.2	12	0.3	5	1.8	10	3.4	5	3.6	3.75	0.5
	5.5	2.3	24.5	0.3	10	0.5	20	4.6	10	1.9	7.5	0.4
	11	0.7	49	0.2	20	0.8	40	0.5	20	1.6	15	0.2
LOQ (µg/mL)		2.6		0.4		11.5		7.2		1.2		0.6
LOD (µg/mL)		0.4		0.1		3.8		2.4		0.4		0.2

**Table 5:** Bioactive components concentration (μg/mL of tea beverage) in green GABA (GGABA) and OGABA extracts. Data are expressed as mean of three independent measurements ± standard deviation. N.D. = no detected.

Compound	GGABA	OGABA
**Flavan-3-ols**		
catechin	361.93 ± 3.9	N.D.
epicatechin	329.80 ± 2.43	33.70 ± 0.11
epicatechin-gallate	208.64 ± 0.65	46.67 ± 0.14
epigallocatechingallate	906.42 ± 0.7	94.81 ± 0.30
**Amino acids**		
GABA	17.81 ± 0.55	22.25 ± 0.65
Glutamic acid	27.10 ± 0.33	15.19 ± 0.45
Glutamine	10.08 ± 0.10	4.39 ± 0.08
Theanine	90.84 ± 0.87	46.15 ± 0.93
gallic acid	N.D.	59.47 ± 0.31
caffeine	1039.85 ± 4.32	525.05 ± 4.28

**Table 6:** Pearson’s linear correlation between the data obtained from antioxidant assays and the data obtained from behavioral tests (DST and TST).

	TBARS	SOD	Cat	GSH
**Swimming**	−0.674 ^a^ *p* < 0.001	0.448 ^a^ *p* = 0.003	0.593 ^a^ *p* < 0.001	0.279 *p* = 0.076
**Climbing**	−0.595 ^a^ *p* < 0.001	0.356 ^b^ *p* = 0.021	0.526 ^a^ *p* < 0.001	0.188 *p* = 0.234
**Immobility (DST)**	0.483 ^a^ *p* = 0.001	−0.271 *p* = 0.088	−0.366 ^b^ *p* = 0.020	−0.020 *p* = 0.901
**Immobility (TST)**	0.568 ^a^ *p* < 0.001	−0.339 ^b^ *p* = 0.029	−0.468 ^a^ *p* = 0.002	−0.129 *p* = 0.415

TBARS: thiobarbituric-reactive substances; SOD: superoxide dismutase activity, Cat: catalase activity; GSH: glutathione. Bivariate Correlations: ^a^ Indicates a correlation at *p* < 0.01.^b^ Indicates a correlation at *p* < 0.05.
